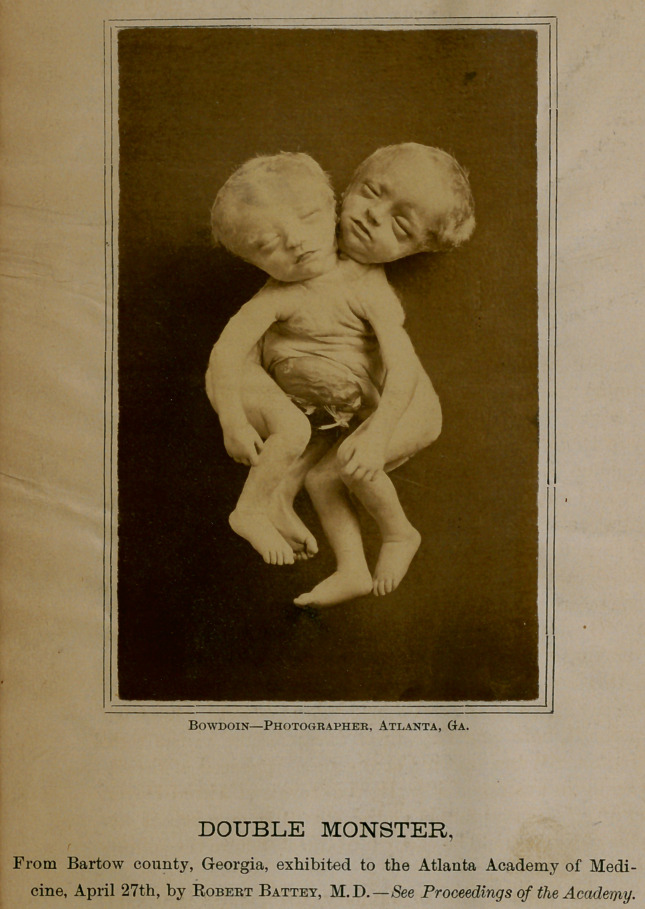# Intestinal Obstructions: A Safe and Ready Method

**Published:** 1874-06

**Authors:** Robert Battey

**Affiliations:** Atlanta, Ga.


ATLANTA

MEDICAL AND SURGICAL JOURNAL.

VOL. XII.] JUNE—1874. [No. 3.

### Original Communications.

### INTESTINAL OBSTRUCTIONS; A Safe and Ready Method.

BY ROBERT BATTEY, M.D., ATLANTA, GA.

*Read before the Atlanta Academy of Medicine.*

The intestinal canal, in man, is susceptible of great distension without injury to its structure or functions. In the *Medical and Surgical Reporter* of 7th March, 1874, it is asserted, upon the authority of Dr. Milbrand, that "very large injections, half a gallon to a gallon, can be administered, by placing a patient upon his elbows and knees, so that the anus becomes the highest point of the intestinal canal;" with the further remark that they are extremely useful in fecal accumulation, intussusception, lesions of the ilio-cæcal valve, etc. The *Medical Record* of January 1st, 1874, extracts from a German journal of recent date the statement that "Gustav Simon has recently demonstrated the possibility of making fluids penetrate the whole length of the large intestine, thereby causing no injury to the part, by means of forced injections per anum. His investigations were conducted on two patients, both suffering from fæcal fistulæ in the right inguinal region. Water injected at the anus appeared at the fistulous opening in five minutes." By the device of Alfred Heger, consisting of an elevated funnel and elastic tube, as much as *five to nine pints* of water could be easily introduced before the sphincter yielded.

The *Gazette Hebdomadaire* of January, 1873, states that Prof. Gustav Simon recommends the introduction of the entire hand into the rectum, and forcing it on into the sigmoid flexure even, for purposes of diagnosis, and asserts that the manipulation can be executed with entire immunity from danger.

As regards the facility with which the four to eight pints of water of Dr. Milbrand, or the five to nine pints of the German operators, can be safely thrown into the larger intestines, their observations are neither new nor so very remarkable. In the August, 1871, number of the *Georgia Medical Companion* the writer contributed a paper upon Enemata, in which it is stated, "Distensile enemata, when judiciously, and, at the same time, *boldly* employed for the removal of intestinal obstructions, can scarcely be over-estimated. The great power of the remedy, coupled with its comparative harmlessness, is not so generally known and utilized as it ought to be. For this purpose the enema must be decidedly unstimulating; it should be even emolient in its properties. Warm water at about the temperature of the blood, is appropriate. The bulk required is ordinarily large, and an abundant supply, at the least *four gallons*, ought to be at hand, that there be no lack. The syringe must be arranged for continuous action, as the modern styles are, and must also be strong and durable; a capacious vessel, likewise, must be in readiness to receive the evacuation, and the bed suitably protected against accident, that it be not soiled.

"The enema is to be administered in the usual manner, the patient being in the obstetrical position, upon the left side. The important issue hanging upon the result fully warrants the placing of the patient so as to be easily accessible to the operator, and even, if he be inexperienced, the exposure of the nates, though an expert will not find the latter expedient either necessary or desirable.

"When complaint is made of the distension, the injection is to be suspended a little, until the feeling of tenesmus passes off, and then resumed slowly and gently, while the patient is urged to retain it as long as possible. When the power of retention is overtaxed and seems about to give way, it ought to be assisted by a folded napkin firmly pressed over the anus, around the tube by the left hand, or better still, by both hands of an assistant. When the combined powers of the patient and assistant will no longer prevent the escape of the fluid, the syringe should be operated rapidly and forcibly for a few moments, until the distress of the patient peremptorily demands a cessation; then, and not until then, is he allowed to get up.

"In the event the obstruction is not removed at the first essay, the patient is allowed a few hours rest, under the tranquilizing influence of opium or chloral, and the effort then renewed whilst he is yet under its influence. Chloroform by inhalation is sometimes required to assist the toleration of the method. It is scarcely necessary to add the precaution that the full power of the distensile enema is never to be evoked *late* in a case of obstruction, or when there is reason to apprehend that the walls of the tube have been materially weakened by resulting inflammation."

In the December, 1871, number of the same periodical the writer contributed another paper, entitled "Intestinal Obstructions," in which a series of cases is reported. In case second a boy, aged ten, received about *twelve pints* of liquid into his intestines on the 28th February, 1868, with prompt relief to a serious intestinal obstruction, believed at the time to be intussusception. In case four, Mr. S., aged 55, received nearly *twenty-four pints*, with prompt relief of intestinal obstruction. In the other cases the quantity of liquid employed was not accurately estimated. It may safely be asserted that it was not less than *twelve to fifteen pints* in any of the adult cases.

The following remarks, appended to the report of cases, are applicable to the purposes of the present paper: "In the first case cited it was manifest, at the post-mortem, that the early use of a distensile enema, of even *three pints* of warm water, would have rescued this patient *promptly* and *certainly*. This inference was irresistible. As it was, the use of purgatives and small purgative enemata in the earlier periods of the illness was continued with a patient pertinacity worthy of a happier result. Repeated partial evacuations were obtained to encourage perseverance in the method adopted, and yet, as the event proved, all to no good end. Why was this so?

"In post-mortem inspections, after intestinal obstruction, it is not unfrequently observed that restricted areas of both the lesser and larger intestines are found to be violently and permanently contracted down into a sort of cord by spasm of the muscular coat, while adjoining portions above and below are of normal or even abnormal distension. It seems quite reasonable to infer that this spasmodic stricturing of the canal is coincident with the severe colicky pains present in the history of the case. While this state of firm contraction exists at any point in the canal, with gaseous distension above and below, if violent peristaltic movements be induced by the exhibition of an active purgative, how readily might the contracted portion slip down and become invaginated into an adjoining dilated part, and intussusception be produced by the purgative dose.

"Again, suppose at some point in the colon a mass of scybala becomes impacted; irritation of the intestine results, colicky pains come on with flatulent distension, while the muscular coat is strongly contracted and firmly grasping the mass in its folds, as in cases five, six and seven. Imagine the action of a vigorous purgative in such circumstances, throwing the intestines into violent peristaltic commotion; how easily might a distended portion above re-duplicate itself over that below, holding the mass in its grasp. Suppose now the three layers overlying the obstructing material, by their three-fold muscular power, should expel it to a point lower down, and leave an intussusception behind. In this case the fecal matters might easily be brought away by purgatives, or purgative enemata, whilst the invaginated intestine is by the same agency, being more and more firmly grasped in muscular spasm, and the fate of the poor sufferer effectually sealed.

"And yet again, suppose a knuckle of intestine has slipped beneath an adventitious band, left by some former local peritonitis and become strangulated. The irritation, the pain, the intestinal muscular spasm which ensues may equally give rise to fatal intussusception, under the stimulus of an active purgative dose.

"Has it been the fortune of the reader to have passed his earlier life upon a Georgia plantation? Has it been his lot to witness in person, in its annually recurring season, the various stages and manipulations of the complex art, popularly known by the term 'hog-killing,' by which the various tissues and organs of that animal are prepared for domestic uses and human sustenance? Has he ever stood over the large tubs of clear spring water, containing the 'sausage skins,' as they are sometimes called, and observed an old negress, who ordinarily conducts the cleansing process, fish up with her finger the end of an intestinal tube, insert a joint of reed into its calibre, and inflate it with her breath? If so, he has taken his first lesson in the management of intestinal obstruction. See how the dead mass of confused shapeless membranes progressively assumes form and semblance as the air distends their parieties. How they suddenly become re-animated, as it were, and rapidly disentangled from the chaotic confusion, wind themselves, like the serpents of Laocoon, in graceful curves about the sable arm of the operator. Observe more closely, as the breath is blown in, and note the propelling power of the inflation; how it causes the intestine to withdraw itself, as though instinctive with real life, from an entanglement aptly comparable to the gordian knot, with all the ease and grace that a serpent emerges from the tangled grass. Take up a loop of the intestine in the hand, confine it between the fingers, and note with what ready power the distending inflation withdraws it from your grasp. *Surely the power of distension from below is the very force needed for the relief of intestinal obstruction* whatever be its cause, if indeed the cause be removable by human art.

"To the distensile enema of simple tepid water, no valid objection can be urged. It is eminently unirritating, nay, soothing and relaxing even, in its properties. It exerts a force directly proportioned to the bulk employed, by reason of the incompressibility of water, a force which can be regulated at will. It is always at hand, and can be successfully administered, if need be, by a hog's bladder and a joint of reed.

"In *every case* of intestinal obstruction, either feared, suspected or known to exist, when the duration does not raise a well grounded apprehension of gangrene, an anodyne having been premised, the distensile enema ought to be the *first*, and for the most part need be the *only* power invoked for the cure. In the treatment of colic and fecal impaction, it is wise to abstain from the use of active purgatives until all spasm of the intestines is allayed, and fecal accumulations are removed by the distensile enema."

Such was the experience, such the views of the writer in November, 1871. It was perfectly evident that water could be passed *per anum*, not only into the rectum and colon, but on through the ilio-cæcal valve into the small intestines; the bulk of liquid passed in showing conclusively that this must have occurred in repeated instances. Subsequently an accidental observation suggested the inquiry, *can the distensile enema be carried through the entire intestinal tract into the stomach and out at the mouth of the living subject?*

Case: Mrs. S., aged twenty-three, married, mother of one child; was seen on the 9th of March, 1873. She had complained of an obscure gastric trouble for near three years; had been for some weeks under medical treatment; had quite persistent vomiting, at times streaked with blood; much gastric pain; bowels obstinately constipated, and the numerous purgatives which had been given her were vomited and the bowels still unrelieved. Her medical attendant regarded the case as one of intestinal obstruction. A new diagnosis of gastric carcinoma was now made, and a copious distensile enema, with the addition of turpentine soap, administered, with the two-fold view of demonstrating the absence of obstruction in the bowels, and at the same time effecting a thorough clearance of the canal. The soapy liquid was slowly but persistently thrown into the rectum, whilst the abdomen was gently kneeded with the hand to encourage the ascent of the liquid to as high a point as practicable. The abdomen became quite distended; eighteen or twenty pints of water had passed into the canal, when a copious vomiting of fluid occurred, followed by a remark from the patient, "Doctor, I taste your soap in my mouth!" and she repeatedly protested that she could not be mistaken.

Case: Mr. H., aged sixty; had double inguinal hernia for many years. He had not unfrequently partial strangulation, but had been accustomed to reduce it himself, without the aid of a physician. On the 4th September, 1873, the hernia of the right side became strangulated, and he reduced it as usual. On the 15th it became strangulated again, but he reduced it in three or four hours. On the 30th strangulated again, and again reduced by himself, but with increased difficulty and after six hours of suffering. The reduction did not, however, on this occasion bring relief to his sufferings as it had done heretofore. He therefore called his physician, Dr. W. L. Selman, of Texas Valley. Finding no relief, doses of castor oil and other purgatives being vomited back and the bowels unmoved, the writer was called in consultation, and requested by Dr. S. to come prepared to open the abdomen and relieve the strangulation should it be found necessary to do so. When seen on the morning of the 4th of October, the fifth day of the last strangulation, his countenance bore an expression of deep anxiety and distress; he was still suffering paroxysms of great pain, in spite of the opiates given, which had, however, afforded him some snatches of sleep during the night. A firm mass could be felt through the abdominal wall, just above the right inguinal ring, and the same mass could be touched by the index finger invaginating the scrotum through the inguinal canal. There was great tenderness complained of when the strangulated mass was touched. The vomiting and obstinate constipation still continued. The patient was chloroformed and simple tepid water injected into the rectum until copious vomitings of discolored fluid occurred, in such quantities as to make it evident that it had passed into the stomach from below. So great was the abdominal distension the water spouted from the anus, when pressure was removed, in a bold stream to a distance quite two feet from the nates, and continued thus to escape until a gallon, perhaps, had been discharged before the power of the sphincter became adequate for its control. On recovering from the anæsthesia he passed the enema in several successive and liberal instalments, with intervals of rest, and accompanied by a satisfactory amount of feces. The relief from pain was prompt and complete; the vomiting ceased at once; a purgative was given, and nothing further was required in the case. Owing to the fact that no suitable arrangements could be had to supply the warm water in quantity at once, it was difficult to make any accurate estimate of the amount used. It must have been little, if anything, short of twenty-four pints.

In January of the present year, the writer, assisted by Prof. J. T. Johnson, experimented upon the cadaver, that the progress of the fluid upwards through the alimentary canal might be watched by the eye. The room was cold, temperature below forty deg.; the abdominal contents and the water employed for injection so cold as to benumb the hands. Notwithstanding these disadvantages, the liquid passed readily along the entire length of the colon, and found no obstacle at the ilio-cæcal valve to its onward progress. Upon reaching the upper portions of the smaller intestine, greater difficulty was encountered on account of the collapsed and matted condition of the cold, cadaveric mass. Movement of the convolutions, however, by the hand, permitted the fluid to still pass onwards until it finally reached the stomach and even flowed out of the mouth upon the table.

Having established, as the writer believes he has satisfactorily done, the fact that a fluid may be entered at the anus and made to permeate the entire intestinal canal, to pass into the stomach, and to be even vomited forth from that organ, in proof of its complete route through the tract, it is proposed to consider briefly the applicability of the *method* to the treatment of the various forms of intestinal obstruction. The classification of Mr.

Erichsen, based upon the inducing cause of the obstruction, will be used for convenience.

1st. Internal hernia; wherein a portion of the gut slips through an aperature in the mesentery, or omentum, or becomes constricted by bands, adhesion, or diverticula, stretching across from one side of the abdomen to the other. In these cases the problem presented in the outset is purely mechanical. Simply withdraw the knuckle of intestine from its imprisonment and the problem is solved. The power which is to accomplish the withdrawal will be illustrated in the bowl of water upon the table. Let some one seize in his fingers the moist intestinal tube, and observe the readiness with which it will be withdrawn from his grasp by the force which distends the tube, and he will at once perceive its applicability to the conditions under consideration. But, suppose muscular spasm should grasp the hernial neck so tightly as to resist effectually the tractile force of the distending fluid. In this case we have the fomenting action of the intestine distended with warm water, and the anæsthetic power of chloroform to relax the spasm. Again, suppose an obstacle be offered by accumulated feces in the strangulated knuckle, too bulky to admit of withdrawal through the constricting ring. In this case we have first the distending force of the liquid to gradually enlarge the ring, and the establishment of a minute stream of water through the incarcerated bowel, softening, disintegrating and gradually washing out the accumulated feces. And yet again, suppose that inflammation has been set up about the neck of the hernia, and exudation of recent lymph has glued the part to the constricting band. It would be difficult to conceive a more safe, and at the same time effective, means of stretching out those bands of adhesion and liberating the imprisoned gut than is afforded by the gentle and steady traction of the distending fluid. Precisely such a stretching out and rupture of soft bands of effused lymph it is believed was accomplished in this way, in the case before cited of Mr. H. This opinion was concurred in by the attending physician, Dr. Selman.

2d. In the case of intussusception, we have likewise, at first, a simple mechanical proposition involved. For its remedy we have a double acting force exerted by the injected water, *i.e.*, the tractile force pulling down the invaginating tube, and the advancing stream of fluid pushing upwards the invaginated mass, or *vice versa*, as the case may be. If there be adhesion formed by bands of recent lymph, the power to gently and surely overcome them has been already noticed.

3d. The ready power of an advancing column of distending fluid to untwist a volvulus needs only to be mentioned to be at once conceded.

4th. In obstruction by malignant disease, when it is proper to exert any force for the enlargement of the constricted portion, the distension by tepid water commends itself, for obvious reasons, in preference to the bougie when the stricture could be thus reached, and of course when its seat could not be reached by the bougie it would possess every desirable advantage.

5th. In intestinal spasm the applicability of the tepid enema with chloroform inhalation has already been alluded to.

6th. Obstruction of the bowel may result from the pressure of a tumor growing near it. In such circumstances the removal of the tumor, when practicable, is the obvious treatment; but when this cannot be accomplished, the hydrostatic force may be used to open a way upwards for the fluid, to soften, to disintegrate and to bring away in diffluent form the excrementitious materials.

7th. Intestinal obstruction may likewise be occasioned by accumulation of indurated feces in impracticable masses. Under such circumstances the distensile enema expands the coats of the intestine, passes up around and above the masses, moistening the scybala, softening them and preparing them for expulsion by peristaltic contractions of the bowel, with the assistance of external manipulation upon the abdomen and the breaking up of the rectal contents.

8th. In considering the diagnosis of a case of intestinal obstruction, the existence of strangulated hernia, whether inguinal or femoral, as the exciting cause, is not to be overlooked. For the relief of strangulated hernia, the ordinary method by taxis, in the great majority of instances, is so successful as to leave nothing to be desired; and yet, in a notable proportion of cases, herniotomy has been found necessary. Very few surgeons, probably, who have had much to do with these cases, have not felt, more or less frequently, the desirableness of a tractile force *within the abdomen* to assist by drawing back the knuckle of intestine whilst they were pushing it in from without. Indeed, such a force has in numerous instances been invoked, in a feeble way it is true, but very effectively in the reduction of cases which would otherwise have been doomed to the knife of the surgeon. The force thus utilized has been simply the gravity of the intestine itself, which has been brought to bear by placing the patient upon the knees, with the chest depressed. In other instances the heels of the patient have been taken over the shoulders of an assistant, whilst the chest and head were left pendant; and others, indeed, have been literally swung up by the heels, with the head downwards. Undoubtedly a most useful force is thus exerted within the body in aid of the surgeon's taxis. In such cases it scarcely needs argument to show that the distensile enema is capable of exerting not only equal, but notably a much greater tractile force than mere gravity. Indeed, the force which may be thus exerted is so potent as to suggest the inquiry, for future experience to determine, *how far it may be relied upon for the reduction of all strangulated hernias, and replace the knife entirely.*

The proposition, successfully carried out, of introducing into the alimentary canal so large a quantity as two and a half, or even three gallons of fluid with safety is believed to be new and original with the writer. But however this may be, the successful passage of fluid throughout the entire tract from anus to mouth, in either the living body or the cadaver, is claimed to be unique and unprecedented. It is by no means assumed, though, that there is anything new in the idea of *forcible distension* of the canal for the relief of intestinal obstruction, or obstipation. This has often been done, and with success. Erichsen states that he has twice succeeded in reducing an intussusception by the injection of air into the rectum, and adds: "When the air is pumped in, it is doubtful whether it passes beyond the ilio-cæcal valve," etc. It has likewise been proposed to introduce into the rectum a solution of bicarbonate of sodium, to be followed by an equivalent solution of some vegetable acid, and to utilize the distending force of the disengaged carbonic acid gas for the relief of obstruction in the gut. This proposal has also been carried out in quite a number of cases with varying results.

It is well known in the majority of households, where the enema is employed as a domestic remedy for constipation, that the entrance of air with the injected fluid produces irritation of the mucous membrane and colicky pains in the bowels. Hence precaution is used to fill the apparatus completely with fluid, to carefully exclude all air, previous to the administration. We constantly observe in the use of anodyne enemata the necessity of a careful exclusion of air, that the enema may be retained and its tranquilizing effects secured. Neglect of this precaution ordinarily ensures the evacuation of the rectum, and when this does not occur, the tranquilizing effect of the opiate is to a great extent antagonized by the irritation set up by even small amounts of air. How unphilosophical then is it to attempt the reduction of an intussusception, an internal or external strangulation, or, indeed, any other form of intestinal obstruction, by a remedy which in its own direct action upon the canal produces irritation, colicky pains and intestinal spasm. How much more rational would it be to employ for this purpose a bland, and even soothing, fluid, such as simple tepid water unquestionably is. This point becomes the more striking when we reflect that one of the leading indications in the treatment is to allay irritation and intestinal spasm, as a means to the liberation of the incarcerated bowel.

Of the distensile force of carbonic acid gas, disengaged by chemical action within the gut itself, it is only necessary to say that it must be most *varying* in amount, with the ever varying composition of the super-carbonates of sodium, whilst the pressure of water is fixed and constant, and susceptible of exact mathematical determination. It is the identical force which causes the heavily soldered seams of a copper soda-water fountain to separate with explosive violence. Of it Dr. Austin Flint, Sr., says: "I have known rupture to result from the injection successively of an acid and alkaline liquid, giving rise to the evolution of gas by combination within the intestine. This method of employing pressure is highly objectionable, because the amount of pressure cannot be regulated."

Is it objected that the hydrostatic pressure of injected water is itself dangerous, when imprudently used, to the integrity of the canal? Unquestionably water may be thrown into the intestine, with a pump of adequate power, in such manner as to rupture the intestinal wall, but it is not to be apprehended that *any danger* of this is incurred by the use of the ordinary elastic-bulb syringe in the hand of the surgeon, carrying a head upon his shoulders, even though the subject be a child. This objection, too, involves, in the very proposition itself, want of ordinary care and prudence, which would condemn most surgical and, indeed, medical, proceedures. It may likewise be said, with point, that in a malady of which Erichsen avers, "It is not only that the surgeon knows that, if the patient be left unrelieved, he must necessarily die, but that he is aware that the *only means* of relief, gastrotomy, is probably *nearly as fatal* as the disease for which it is undertaken "—such objection is captious in character and merits no further answer.

In speaking of intussusception, Dr. Flint remarks, in his work upon Practice, " If practicable the injection should be made through a long flexible tube, carried into the intestine as far as it can be made to pass without undue force. The object is to effect the restoration by the upward pressure of the air or water, the invagination being, in the great majority of cases, in a downward direction. The injections are not to be pushed beyond the point at which they are borne without much suffering, and, if they do not succeed after a fair trial, they are not to be persisted in. They will very rarely succeed after the invaginated portion of intestine has become swolen by congestion and the peritoneal surfaces in contact have become adherent. If pushed too far, rupture of the intestine below the seat of the obstruction may be produced. * * * These measures for reduction are, of course, of no avail if the seat of the invagination be above the ilio-cæcal valve."

It seems, indeed, astounding that the profession should so long hold to the fallacious idea that liquids, and especially so bland and unirritating a liquid as tepid water, can be carried to a higher point in the intestinal canal by means of O'Burne's elastic tube passed into the colon, to any extent, however high up. It would appear that a little reflection would make it clear, 1st, That a liquid, perfectly mobile in all its parts, pressing equally in every direction, tending to insinuate itself into every crevice, readily entering even the minute pores of wood, and, under suitable mechanical appliances, capable of exerting the stupendous, and at the same time perfectly manageble powers of the hydraulic press, must insinuate itself readily anywhere in the alimentary canal that O'Burne's tube could possibly pass, and, indeed, far beyond the attainable reach of that instrument. 2d. That this penetration can be effected with superior safety by a liquid endowed with unerring power of instinctive selection, to search out the pervious point of a tortuous and convoluted canal, of which power the O'Burne tube, directed by the most skilled hand, must be acknowledged to be wholly incapable.

When it is considered that the remarks and caution quoted from Dr. Flint are directed to a most formidable malady, of which he himself assures us, "The prognosis is extremely unfavorable. The usual mode of recovery in the exceptional cases in which the affection does not end fatally, has been stated, viz.: by sloughing away of the invaginated portion of intestine, the adhesions at the point of entrance being permanent,"—it seems indeed strange that so much stress should be laid upon the contingent danger of damage by over zeal in the application of so promising a remedy as the distensile enema most certainly is. This apparent inconsistency is explainable only by the inference that that learned, able and conservative author has not as yet developed in his experience the power and scope and comparative safety of the method in question. Evidently he is ignorant of the extent to which water may be made to traverse the canal without any undue violence to any of its parts. Upon this point, too, it is certainly legitimate, nay, it is the bounden duty, of the practitioner, in so desperate a strait, to well and truly weigh the magnitude of the issue at stake, the extreme peril of his patient upon the one hand, with the dangers which may attend upon his remedial measures, and the hopefulness of their success upon the other hand, and, by virtue of his authority and enlightened judgment, to declare absolutely what is to be done in the circumstances before him. Surely in such case it ought not, it cannot be demanded of him that he shall restrict his ministrations to such measures as shall be *absolutely free from dangerous consequences.* It is enough that the peril of the condition under treatment shall far outweigh any hazard which may be contingent upon the proposed remedy.

An earnest, but respectful, protest must be entered against the injunction that "the injections are not to be pushed beyond the point at which they are borne without much suffering." The statement—"they will very rarely succeed after the invaginated portion of intestine has become swolen by congestion and the peritoneal surfaces in contact have become adherent"—must be called in question. Obedience to the injunction will most effectually rob us of a power for good in a very large class of most desperate and otherwise well nigh hopeless cases. A blind acceptance of the statement will as effectually paralyze our efforts in the crisis when valuable lives turn upon our decision. Any comparison between the proposed method and gastrotomy for the relief of intestinal obstructions would be a work of supererogation. The simplicity and comparative freedom from all danger in the one,

BOWDOIN—PHOTOGRAPHER, ATLANTA, GA.

### DOUBLE MONSTER,

From Bartow county, Georgia, exhibited to the Atlanta Academy of Medicine, April 27th, by ROBERT BATTEY, M.D.—*See Proceedings of the Academy.*

would unquestionably dictate its thorough trial before the other could be contemplated.

In conclusion, the writer desires to express the sentiments of reverence and esteem in which he holds the *fathers in medicine,* both living and dead. While he is disposed to yield to no man precedence in doing honor to them and their distinguished labors, he would as unhesitatingly declare his utter aversion to that abject slavery to the mere prestige of authority which tends to check all progress in medical science. Every case of disease which presents itself for remedy has its own individual peculiarities, and must be considered as an integer to itself. The practitioner upon whom devolves the management of the case must decide its points under the illumination of his own mental light, even though this be but a rush-light. He who blindly follows the mere dictum of an authority, is in no sense a *physician.*

## Figures and Tables

**Figure f1:**